# Supplementary figures and images for: RNA-binding proteins direct myogenic cell fate decisions
Source: eLife. 2022 Jun 13;11:e75844. doi: 10.7554/eLife.75844 (PMC9191894; doi:10.7554/eLife.75844)

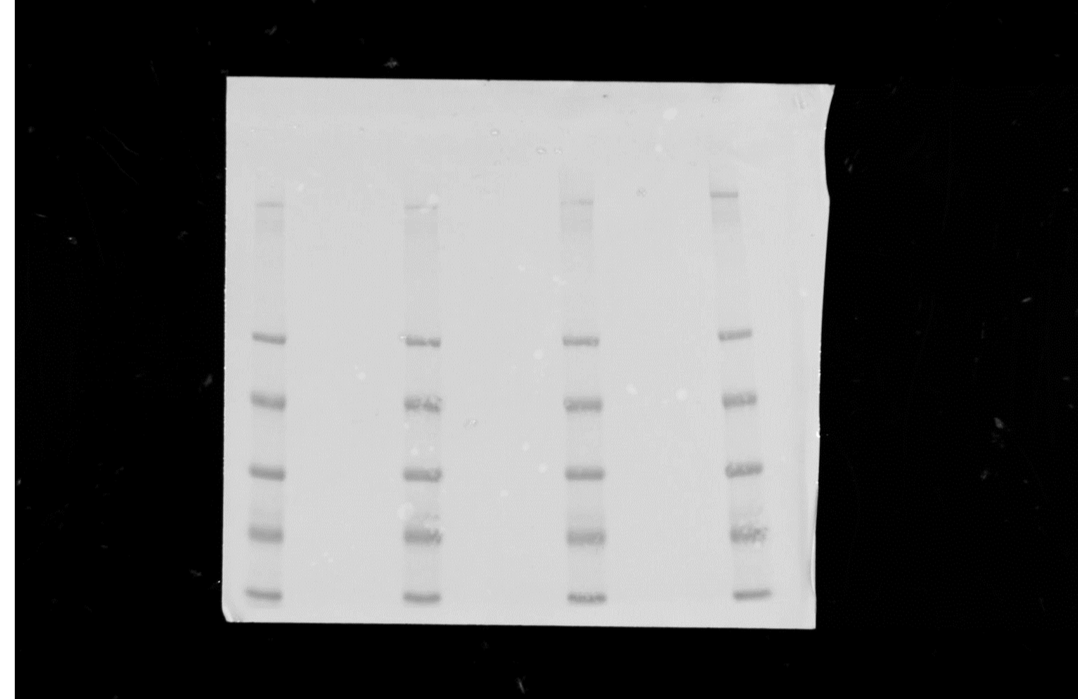

Supplement: Figure 3—figure supplement 1—source data 1. — Raw and annotated Western blot image of Hnrnpa2b1 and Gapdh protein expression from whole uninjured (UI) and 5 days post injury mouse tibialis anterior muscle. [file elife-75844-fig3-figsupp1-data1.zip › Figure S3 - Source data/Fig_S3B_blot_ladder_raw.png]

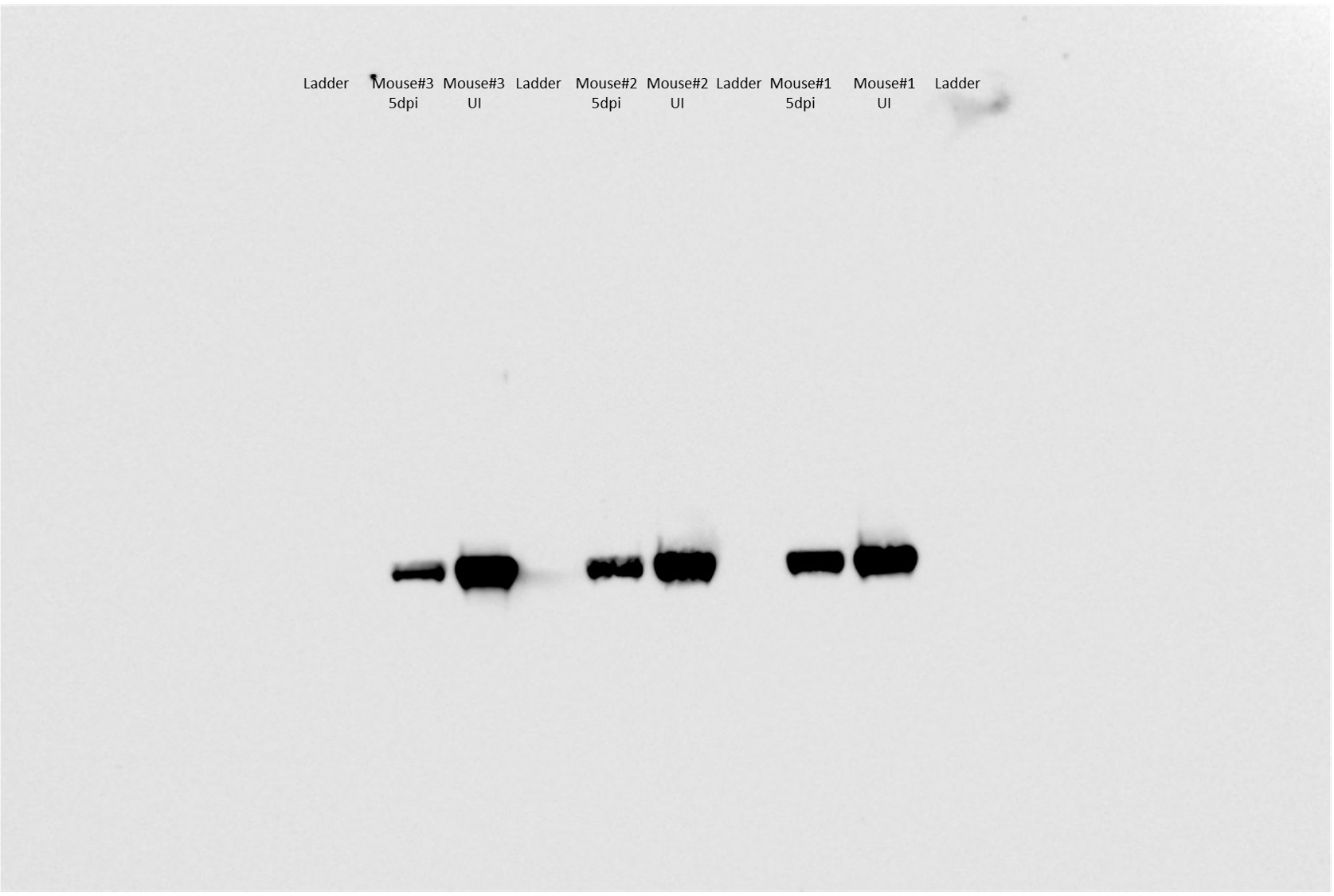

Supplement: Figure 3—figure supplement 1—source data 1. — Raw and annotated Western blot image of Hnrnpa2b1 and Gapdh protein expression from whole uninjured (UI) and 5 days post injury mouse tibialis anterior muscle. [file elife-75844-fig3-figsupp1-data1.zip › Figure S3 - Source data/Fig_S3B_GAPDH_blot_annotated.png]

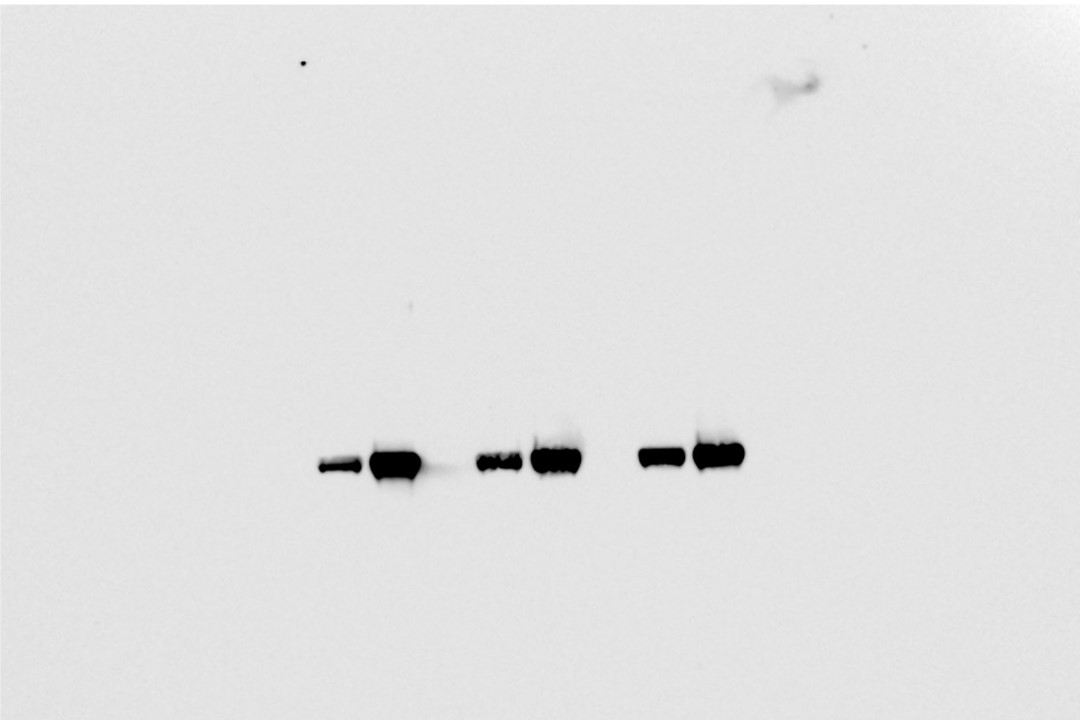

Supplement: Figure 3—figure supplement 1—source data 1. — Raw and annotated Western blot image of Hnrnpa2b1 and Gapdh protein expression from whole uninjured (UI) and 5 days post injury mouse tibialis anterior muscle. [file elife-75844-fig3-figsupp1-data1.zip › Figure S3 - Source data/Fig_S3B_GAPDH_blot_raw.jpg]

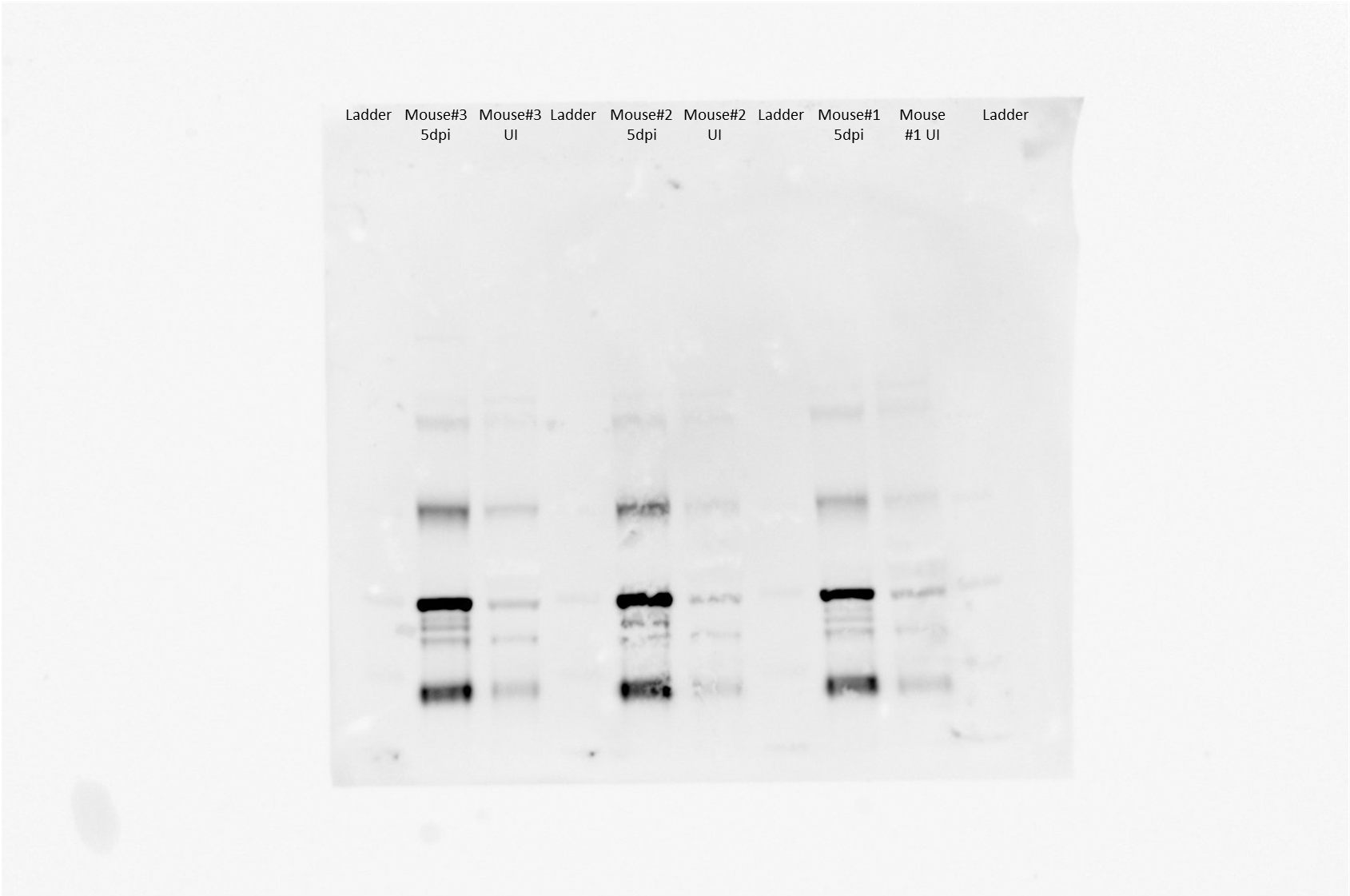

Supplement: Figure 3—figure supplement 1—source data 1. — Raw and annotated Western blot image of Hnrnpa2b1 and Gapdh protein expression from whole uninjured (UI) and 5 days post injury mouse tibialis anterior muscle. [file elife-75844-fig3-figsupp1-data1.zip › Figure S3 - Source data/Fig_S3B_Hnrnpa2b1_blot_annotated.png]

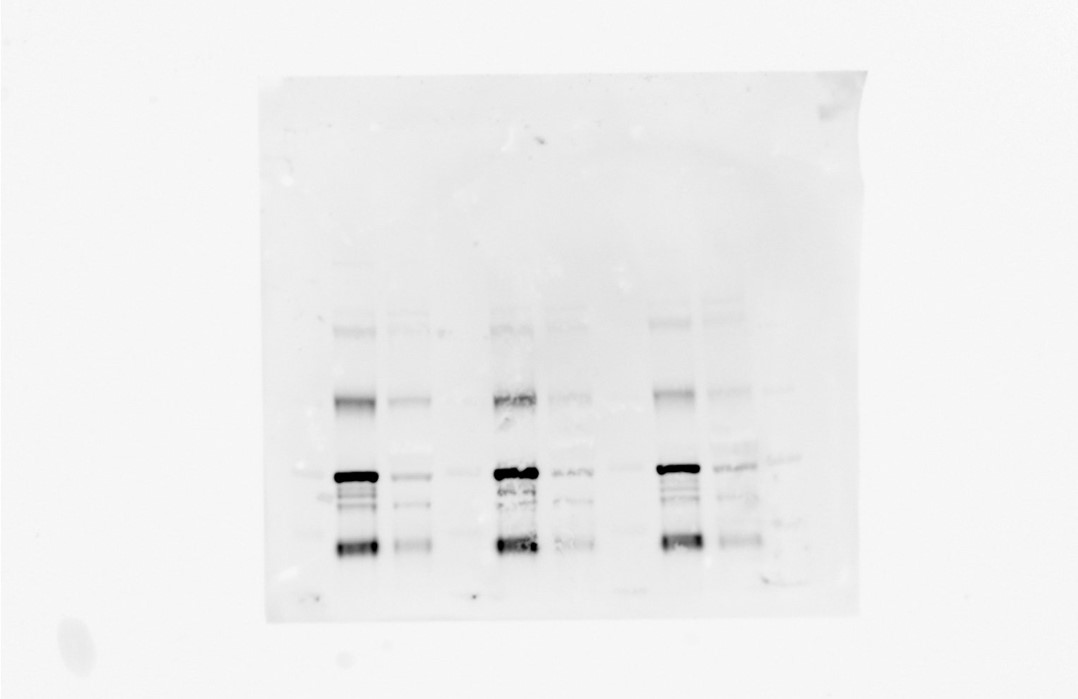

Supplement: Figure 3—figure supplement 1—source data 1. — Raw and annotated Western blot image of Hnrnpa2b1 and Gapdh protein expression from whole uninjured (UI) and 5 days post injury mouse tibialis anterior muscle. [file elife-75844-fig3-figsupp1-data1.zip › Figure S3 - Source data/Fig_S3B_Hnrnpa2b1_blot_raw.jpg]

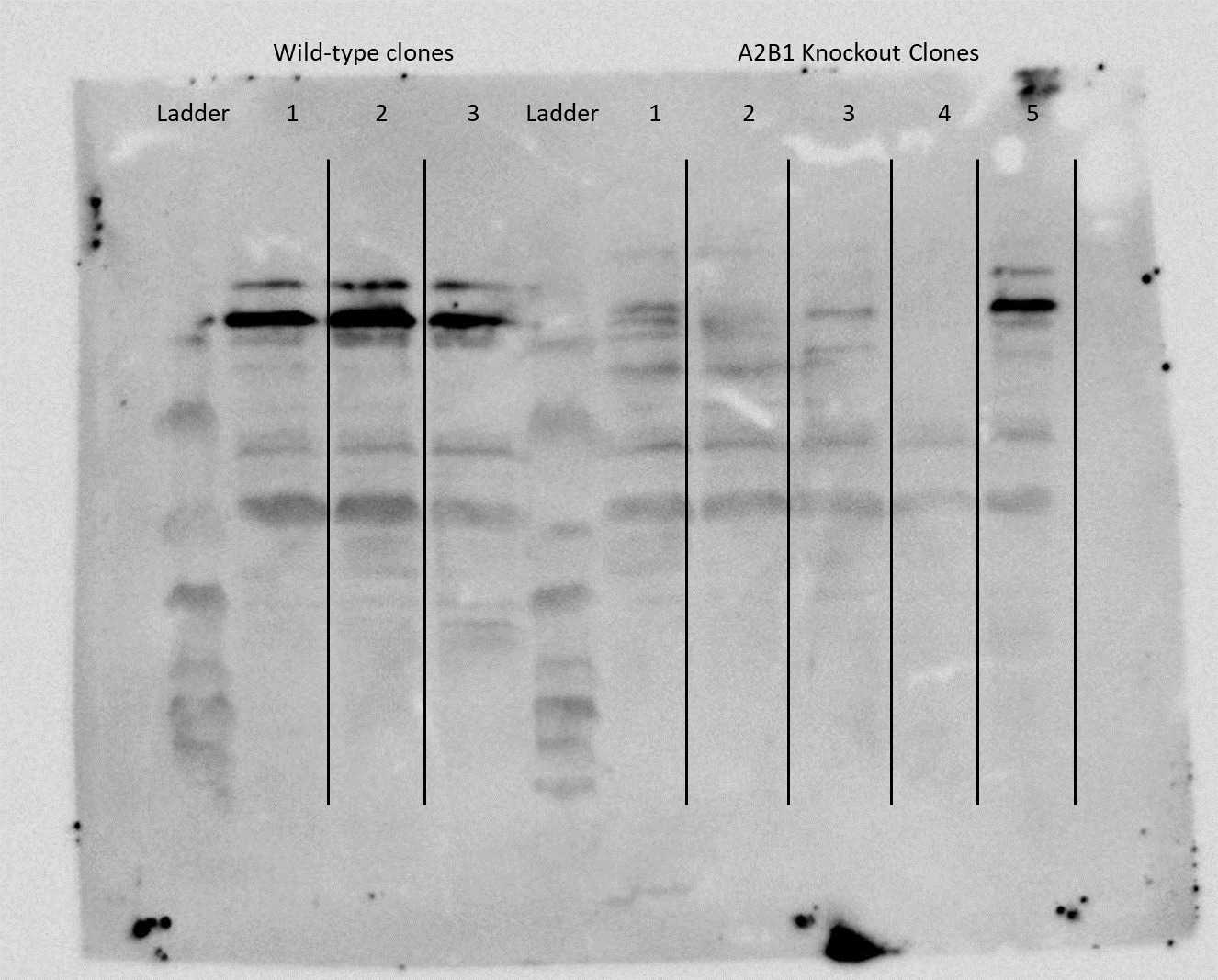

Supplement: Figure 4—source data 1. — Raw and annotated Western blot image of Hnrnpa2b1 and Gapdh in wild type (WT) and Hnrnpa2b1 knockout (KO C2C12 myoblasts). [file elife-75844-fig4-data1.zip › Figure 4 and S4 - Source data/S4C_A2B1_annotated_blot.png]

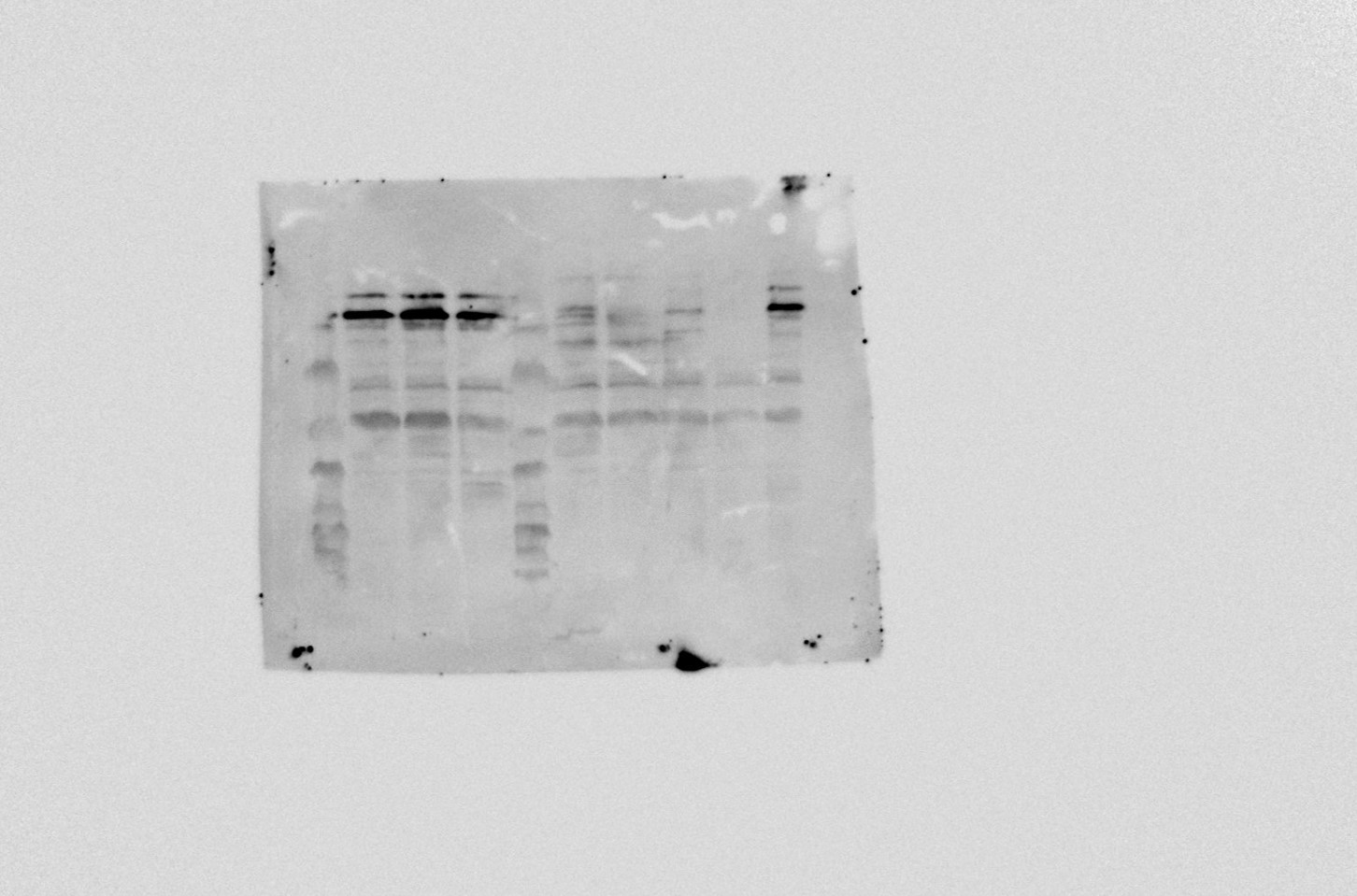

Supplement: Figure 4—source data 1. — Raw and annotated Western blot image of Hnrnpa2b1 and Gapdh in wild type (WT) and Hnrnpa2b1 knockout (KO C2C12 myoblasts). [file elife-75844-fig4-data1.zip › Figure 4 and S4 - Source data/S4C_A2B1_raw_blot.jpg]

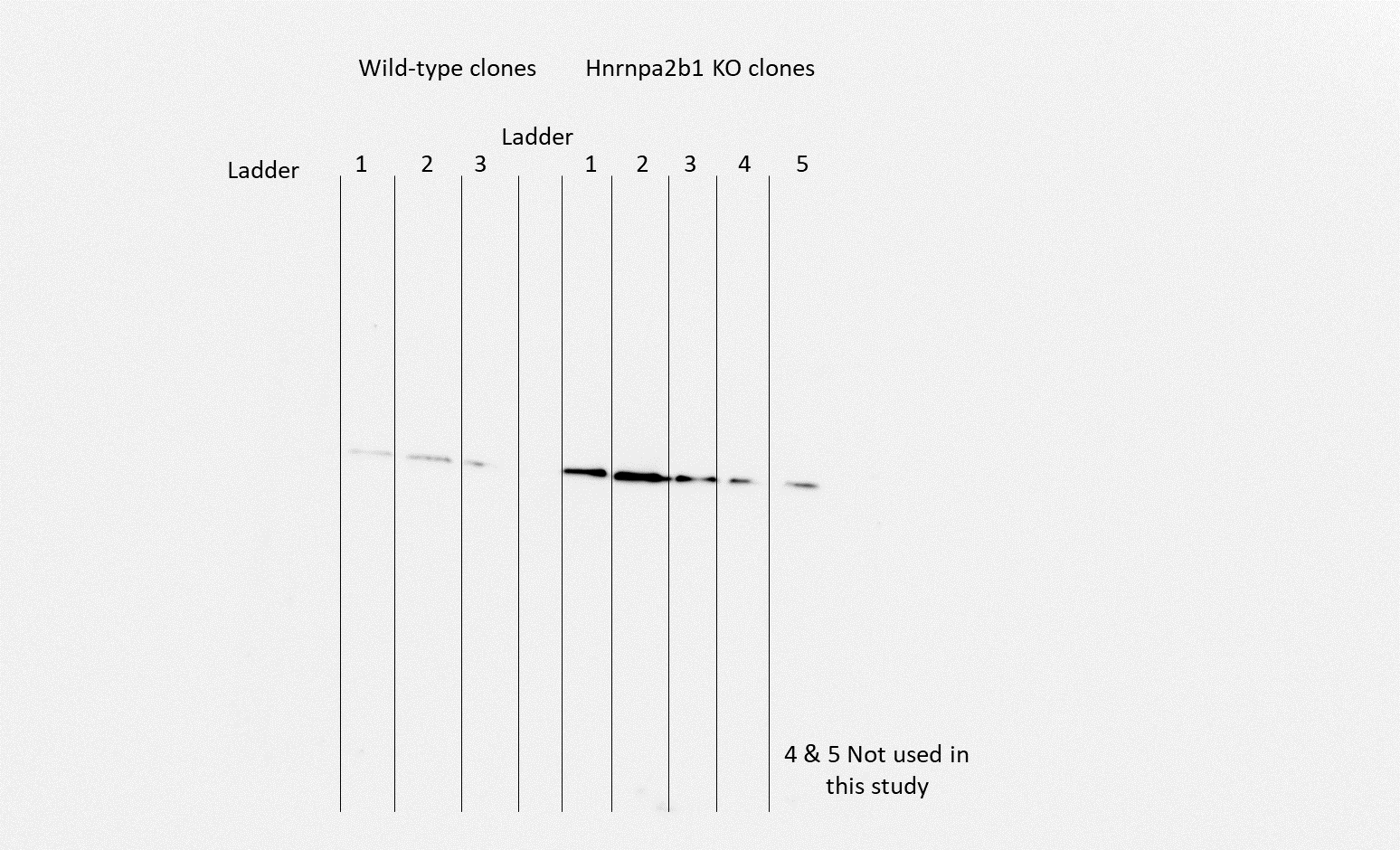

Supplement: Figure 4—source data 1. — Raw and annotated Western blot image of Hnrnpa2b1 and Gapdh in wild type (WT) and Hnrnpa2b1 knockout (KO C2C12 myoblasts). [file elife-75844-fig4-data1.zip › Figure 4 and S4 - Source data/S4C_GAPDH_annotated_blot.png]

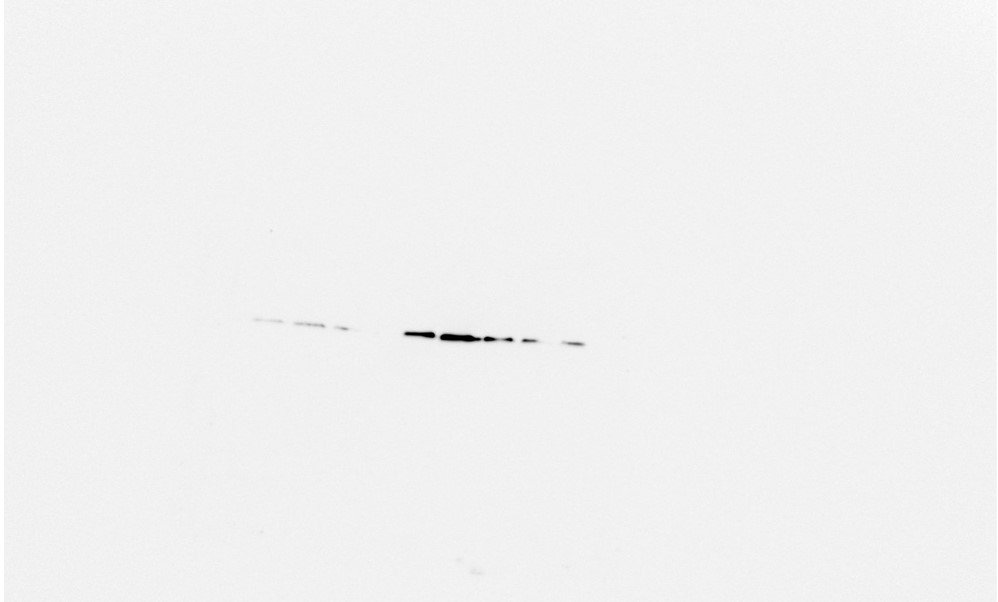

Supplement: Figure 4—source data 1. — Raw and annotated Western blot image of Hnrnpa2b1 and Gapdh in wild type (WT) and Hnrnpa2b1 knockout (KO C2C12 myoblasts). [file elife-75844-fig4-data1.zip › Figure 4 and S4 - Source data/S4C_GAPDH_raw_blot.jpg]

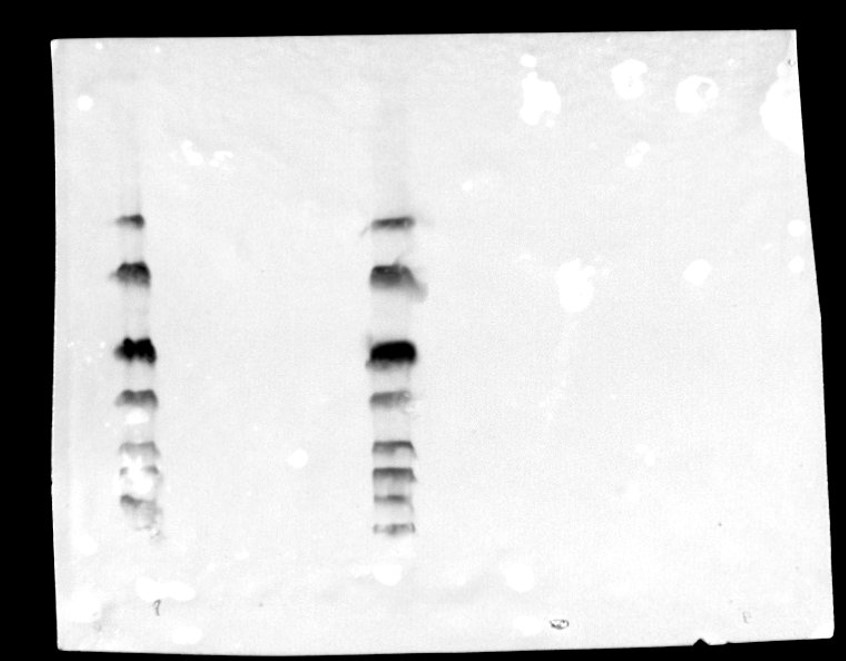

Supplement: Figure 4—source data 1. — Raw and annotated Western blot image of Hnrnpa2b1 and Gapdh in wild type (WT) and Hnrnpa2b1 knockout (KO C2C12 myoblasts). [file elife-75844-fig4-data1.zip › Figure 4 and S4 - Source data/S4C_ladder_raw.jpg]

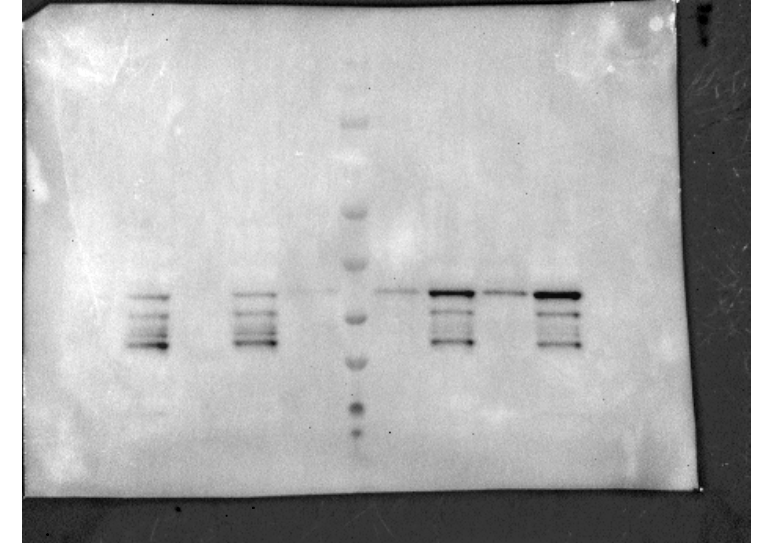

Supplement: Figure 6—figure supplement 1—source data 1. — (A) Raw and annotated blot of autoradiogram of 32P-labeled Hnrnpa2b1 RNA–RNA complexes fractionated by PAGE (B) and (C) immunoprecipitation of Hnrnpa2b1 RNA complexes used for enhanced UV crosslinking and immunoprecipitation (eCLIP) in C2C12 myoblasts or myotubes (n = 2 biologically independent samples). [file elife-75844-fig6-figsupp1-data1.zip › Figure S6 - Source data/FigS6B_blot_raw.tif]

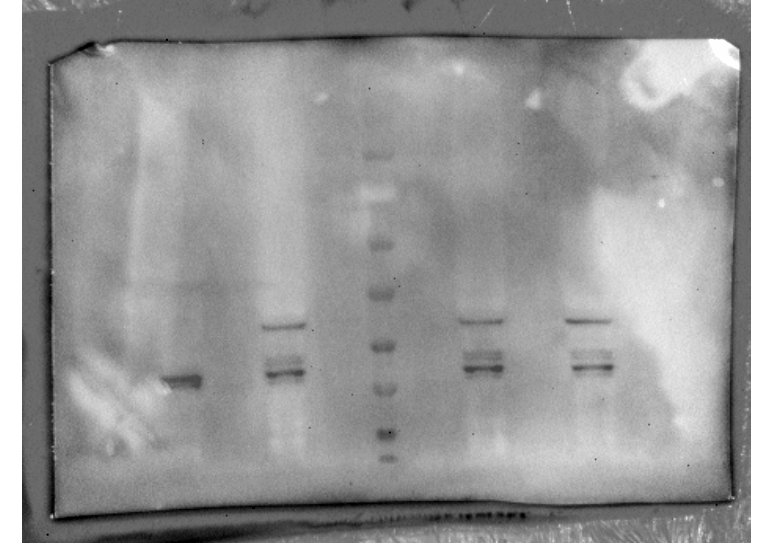

Supplement: Figure 6—figure supplement 1—source data 1. — (A) Raw and annotated blot of autoradiogram of 32P-labeled Hnrnpa2b1 RNA–RNA complexes fractionated by PAGE (B) and (C) immunoprecipitation of Hnrnpa2b1 RNA complexes used for enhanced UV crosslinking and immunoprecipitation (eCLIP) in C2C12 myoblasts or myotubes (n = 2 biologically independent samples). [file elife-75844-fig6-figsupp1-data1.zip › Figure S6 - Source data/FigS6C_blot_raw.tif]

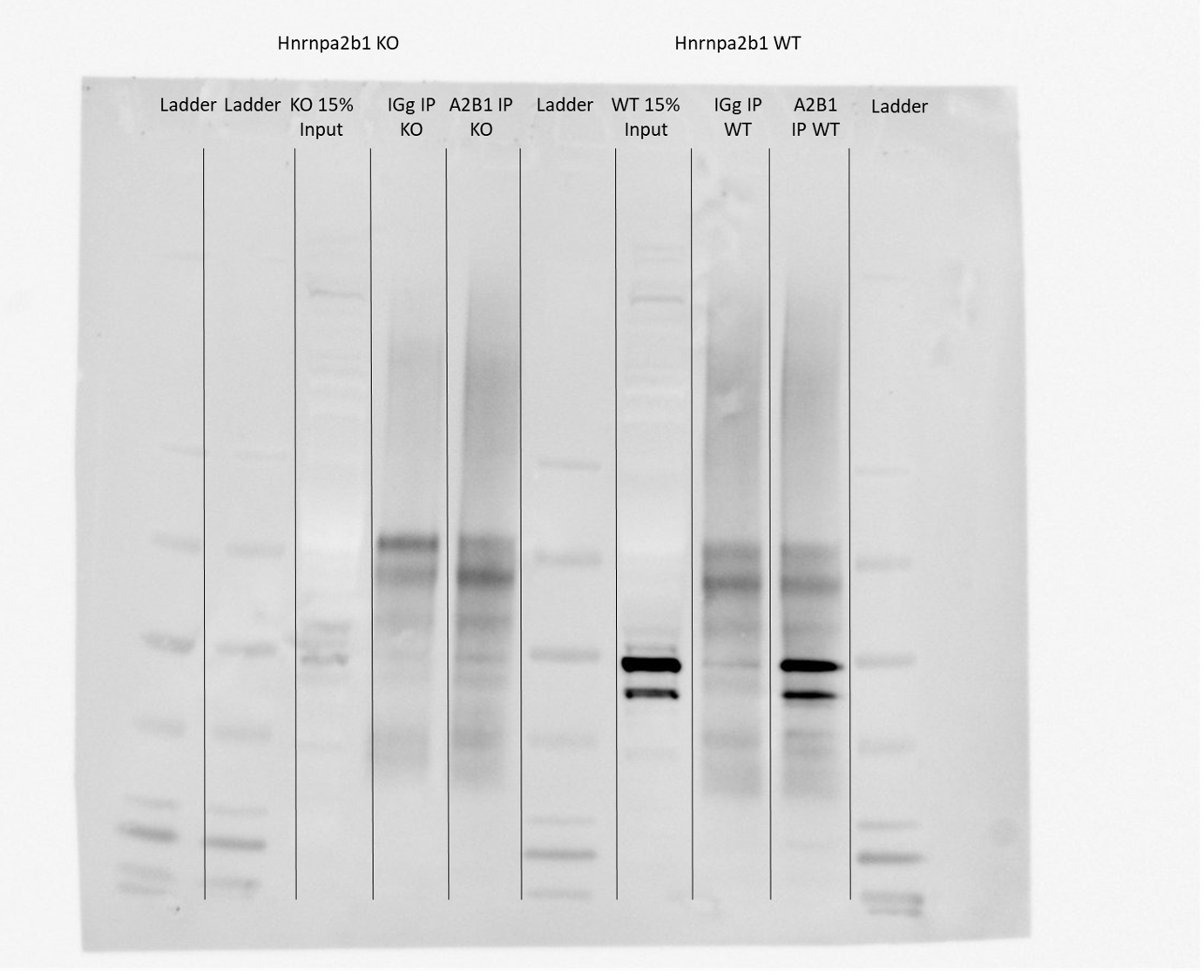

Supplement: Figure 6—figure supplement 1—source data 1. — (A) Raw and annotated blot of autoradiogram of 32P-labeled Hnrnpa2b1 RNA–RNA complexes fractionated by PAGE (B) and (C) immunoprecipitation of Hnrnpa2b1 RNA complexes used for enhanced UV crosslinking and immunoprecipitation (eCLIP) in C2C12 myoblasts or myotubes (n = 2 biologically independent samples). [file elife-75844-fig6-figsupp1-data1.zip › Figure S6 - Source data/FigS6H_Hnrnpa2b1blot_annotated.png]

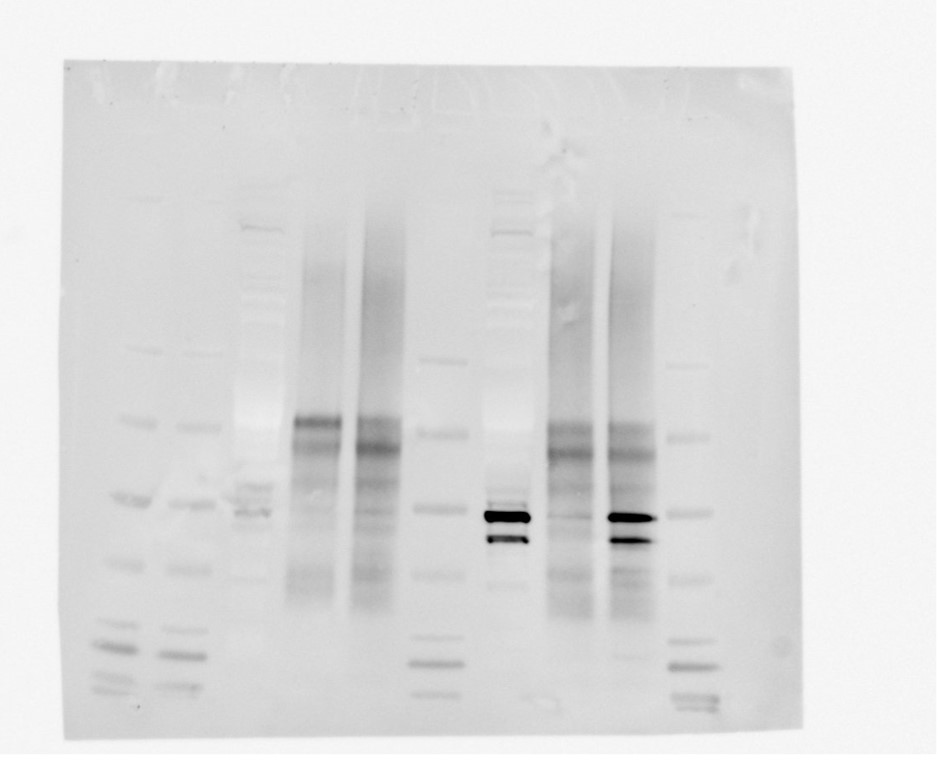

Supplement: Figure 6—figure supplement 1—source data 1. — (A) Raw and annotated blot of autoradiogram of 32P-labeled Hnrnpa2b1 RNA–RNA complexes fractionated by PAGE (B) and (C) immunoprecipitation of Hnrnpa2b1 RNA complexes used for enhanced UV crosslinking and immunoprecipitation (eCLIP) in C2C12 myoblasts or myotubes (n = 2 biologically independent samples). [file elife-75844-fig6-figsupp1-data1.zip › Figure S6 - Source data/FigS6H_Hnrnpa2b1blot_raw.png.jpg]

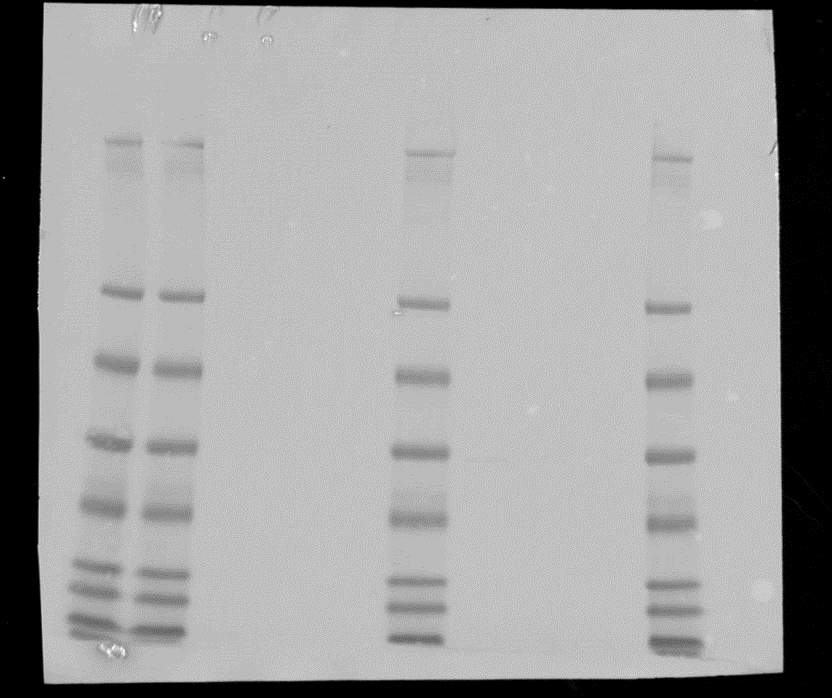

Supplement: Figure 6—figure supplement 1—source data 1. — (A) Raw and annotated blot of autoradiogram of 32P-labeled Hnrnpa2b1 RNA–RNA complexes fractionated by PAGE (B) and (C) immunoprecipitation of Hnrnpa2b1 RNA complexes used for enhanced UV crosslinking and immunoprecipitation (eCLIP) in C2C12 myoblasts or myotubes (n = 2 biologically independent samples). [file elife-75844-fig6-figsupp1-data1.zip › Figure S6 - Source data/FigS6H_ladder_raw.png]
