# Supplementary material for: RNA-binding proteins direct myogenic cell fate decisions
Source: eLife. 2022 Jun 13;11:e75844. doi: 10.7554/eLife.75844 (PMC9191894; doi:10.7554/eLife.75844)
Supplement: Figure 6—figure supplement 1—source data 1. — (A) Raw and annotated blot of autoradiogram of 32P-labeled Hnrnpa2b1 RNA–RNA complexes fractionated by PAGE (B) and (C) immunoprecipitation of Hnrnpa2b1 RNA complexes used for enhanced UV crosslinking and immunoprecipitation (eCLIP) in C2C12 myoblasts or myotubes (n = 2 biologically independent samples). [file elife-75844-fig6-figsupp1-data1.zip › Figure S6 - Source data/FigS6A_32p-labeled RNA annotated.pdf]

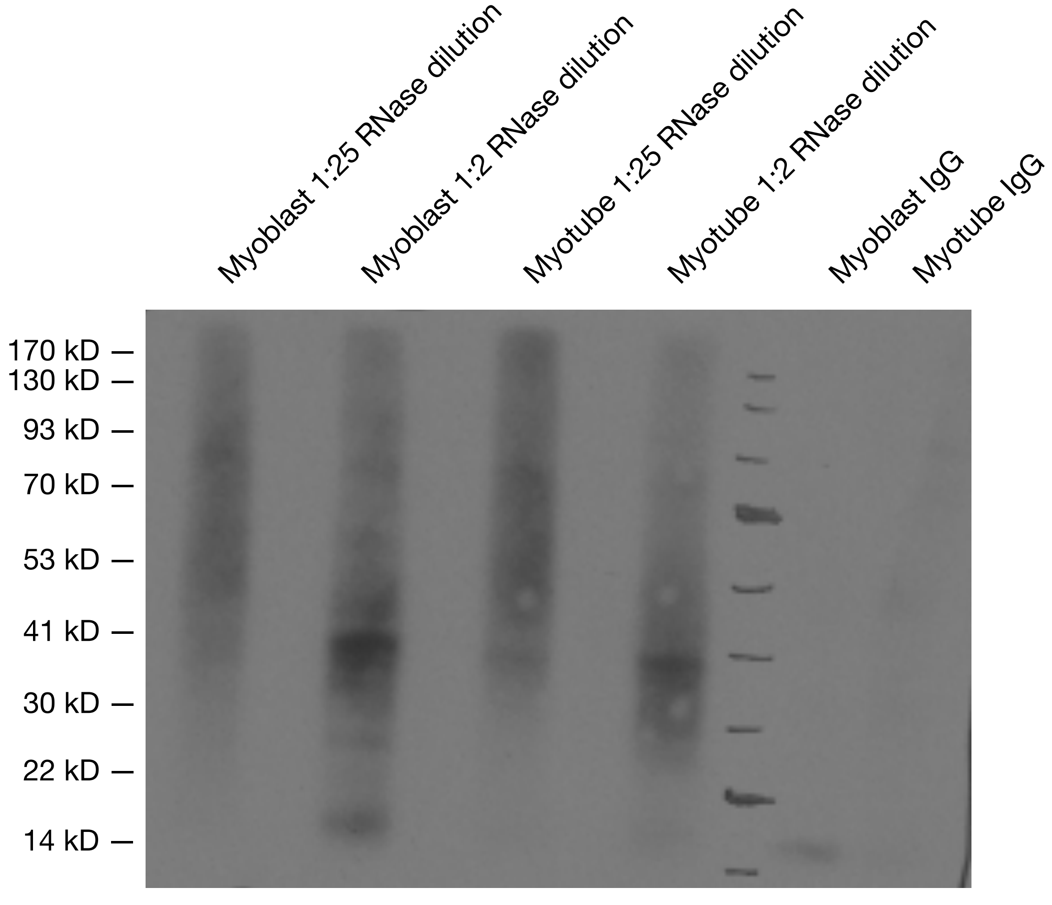
Myoblast 1:25 RNase dilution
Myoblast 1:2 RNase dilution
Myotube 1:25 RNase dilution
Myotube 1:2 RNase dilution
Myoblast IgG
Myotube IgG
170 kD
130 kD
93 kD
70 kD
53 kD
41 kD
30 kD
22 kD
14 kD
