# Supplementary material for: RNA-binding proteins direct myogenic cell fate decisions
Source: eLife. 2022 Jun 13;11:e75844. doi: 10.7554/eLife.75844 (PMC9191894; doi:10.7554/eLife.75844)
Supplement: Figure 6—figure supplement 1—source data 1. — (A) Raw and annotated blot of autoradiogram of 32P-labeled Hnrnpa2b1 RNA–RNA complexes fractionated by PAGE (B) and (C) immunoprecipitation of Hnrnpa2b1 RNA complexes used for enhanced UV crosslinking and immunoprecipitation (eCLIP) in C2C12 myoblasts or myotubes (n = 2 biologically independent samples). [file elife-75844-fig6-figsupp1-data1.zip › Figure S6 - Source data/FigS6B_Myoblast annotated.pdf]

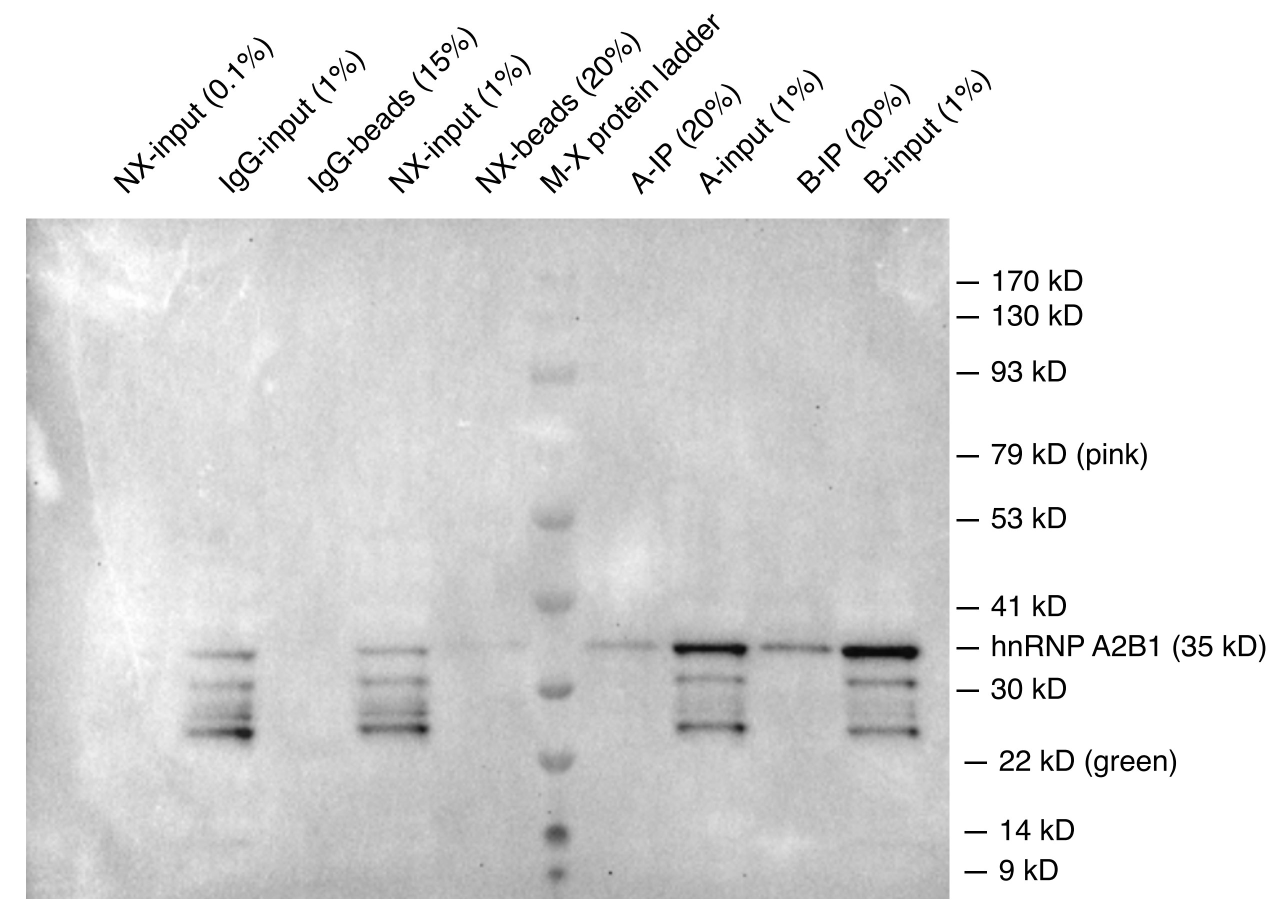
NX-input (0.1%)
IgG-input (1%)
IgG-beads (15%)
NX-input (1%)
NX-beads (20%)
M-X protein ladder
A-IP (20%)
A-input (1%)
B-IP (20%)
B-input (1%)
170 kD
130 kD
93 kD
79 kD (pink)
53 kD
41 kD
hnRNP A2B1 (35 kD)
30 kD
22 kD (green)
14 kD
9 kD
